# 606. Implementation of a Telehealth-based OPAT Early Post-Discharge Clinic May Reduce Hospital Readmission

**DOI:** 10.1093/ofid/ofab466.804

**Published:** 2021-12-04

**Authors:** Nicolas W Cortes-Penfield, Nicolas W Cortes-Penfield, Melissa LeMaster, Bryan Alexander

**Affiliations:** 1 University of Nebraska Medical Center, Omaha, NE; 2 Nebraska Medicine, Omaha, Nebraska

## Abstract

**Background:**

Recent studies suggest that early post-discharge follow-up for patients receiving outpatient parenteral antimicrobial therapy (OPAT) reduces readmission rates. We report our experience implementing a telehealth-based clinic to facilitate early (1-2 week) follow-up for selected OPAT patients perceived to be at high risk for readmission.

**Methods:**

We identified patients who met criteria for and completed a supplemental OPAT telehealth visit following the initial seven months after implementation of this clinic (11/1/20 – 5/31/21). Clinical criteria triggering intake of patients for these visits included: endovascular or cardiac device-related infection; treatment with vancomycin, oxacillin/nafcillin, or aminoglycosides; ≥2 prior hospitalizations within past 1 year; treating Infectious Disease or OPAT team’s subjective assessment of high readmission risk. Patients planned for < 14 days of OPAT therapy were excluded. Categorical variables were compared using a Chi-square test at the α=0.05 level of significance.

**Results:**

A total of 49 patients completed a telehealth visit; mean time from discharge to telehealth visit was 12.1 days (SD +/- 3.9). An intervention was made in 27% of these visits (13 of 49 patients), most commonly involving attempted mitigation of an adverse event or line-related complication (7 cases). The all-cause, 30-day readmission rate for this cohort was 6.1% (3 of 49 patients), while the rate for OPAT patients who did not receive an early telehealth visit during the same period was 22.7% (52 of 229 patients) which was statistically significant (p=0.008). This association of benefit was also found when comparing infection-related, 30-day readmission rates (0% vs 7.4%, p=0.049).

**Conclusion:**

Implementation of OPAT telehealth encounters for high-risk patients resulted in a high rate of intervention to mitigate adverse events of OPAT therapy. Readmission occurred less than one-third as frequently in the telehealth group compared to patients with no early follow-up visit. Telehealth-based encounters appear comparable in effectiveness to those previously reported utilizing in-person visits, introducing efficiencies that may allow for broader implementation of this intervention.

**Disclosures:**

**Nicolas W. Cortes-Penfield, MD**, Nothing to disclose **Bryan Alexander, PharmD**, **Astellas Pharma** (Advisor or Review Panel member)

